# Serotonin Alters the Phase Equilibrium of a Ternary Mixture of Phospholipids and Cholesterol

**DOI:** 10.3389/fphys.2020.578868

**Published:** 2020-10-23

**Authors:** Oskar Engberg, Anna Bochicchio, Astrid F. Brandner, Ankur Gupta, Simli Dey, Rainer A. Böckmann, Sudipta Maiti, Daniel Huster

**Affiliations:** ^1^Institute for Medical Physics and Biophysics, University of Leipzig, Leipzig, Germany; ^2^Computational Biology, Department of Biology, Friedrich-Alexander University Erlangen-Nürnberg, Erlangen, Germany; ^3^Department of Chemical Sciences, Tata Institute of Fundamental Research, Mumbai, India

**Keywords:** ^2^H NMR spectroscopy, molecular dynamics simulation, domain size, raft mixture, line tension

## Abstract

Unsaturated and saturated phospholipids tend to laterally segregate, especially in the presence of cholesterol. Small molecules such as neurotransmitters, toxins, drugs etc. possibly modulate this lateral segregation. The small aromatic neurotransmitter serotonin (5-HT) has been found to bind to membranes. We studied the lipid structure and packing of a ternary membrane mixture consisting of palmitoyl-oleoyl-phosphatidylcholine, palmitoyl-sphingomyelin, and cholesterol at a molar ratio of 4/4/2 in the absence and in the presence of 5-HT, using a combination of solid-state ^2^H NMR, atomic force microscopy, and atomistic molecular dynamics (MD) simulations. Both NMR and MD report formation of a liquid ordered (L_*o*_) and a liquid disordered (L_*d*_) phase coexistence with small domains. Lipid exchange between the domains was fast such that single component ^2^H NMR spectra are detected over a wide temperature range. A drastic restructuring of the domains was induced when 5-HT is added to the membranes at a 9 mol% concentration relative to the lipids. ^2^H NMR spectra of all components of the mixture showed two prominent contributions indicative of molecules of the same kind residing both in the disordered and the ordered phase. Compared to the data in the absence of 5-HT, the lipid chain order in the disordered phase was further decreased in the presence of 5-HT. Likewise, addition of serotonin increased lipid chain order within the ordered phase. These characteristic lipid chain order changes were confirmed by MD simulations. The 5-HT-induced larger difference in lipid chain order results in more pronounced differences in the hydrophobic thickness of the individual membrane domains. The correspondingly enlarged hydrophobic mismatch between ordered and disordered phases is assumed to increase the line tension at the domain boundary, which drives the system into formation of larger size domains. These results not only demonstrate that small membrane binding molecules such as neurotransmitters have a profound impact on essential membrane properties. It also suggests a mechanism by which the interaction of small molecules with membranes can influence the function of membrane proteins and non-cognate receptors. Altered membrane properties may modify lateral sorting of membrane protein, membrane protein conformation, and thus influence their function as suspected for neurotransmitters, local anesthetics, and other small drug molecules.

## Introduction

Small molecules of a molecular weight of less than 1 kDa play key roles in various important processes in biology as receptor agonists or antagonists, (neuro)transmitters, toxins, defense agents against predation and many others (Stockwell, 2004). Also, most pharmaceuticals are small molecules (Scott et al., 2016). Typically, the mode of action of these compounds is well described by orthosteric binding to their target proteins (Weis and Kobilka, 2018). More recently, also allosteric regulation pathways leading to functional selectivity have been described through which small molecules interact with their targets (Macpherson and Anastasiou, 2017). Both biologically and pharmacologically active compounds are often lipophilic and, especially when administered in relatively high dose that can reach a few mol% (Bruns et al., 2000), may unspecifically interact with the cellular membrane (Postila et al., 2016; Josey et al., 2020; Turnbulla et al., 2020). Binding of small lipophilic molecules to membranes is described by a partition equilibrium and can lead to substantial accumulation of the respective compound. In this way, the membrane represents a reservoir for these molecules. As the dimensionality of the diffusion of these compounds is reduced from three in solution to two on the membrane surface, membrane partitioning of small molecules leads to an increase in their effective concentration by a factor of ∼1,000 which is increasing the probability of binding to a cognate receptors (Sargent and Schwyzer, 1986; Brunsveld et al., 2009; Posttila and Róg, 2020).

Small molecules such as neurotransmitters often strongly interact with membranes and lipids (Peters et al., 2013; Postila et al., 2016; Josey et al., 2020; Posttila and Róg, 2020). This has several important consequences as lipids are involved in many physiological processes such as the regulation of synapse development and plasticity, presynaptic vesicle release, regulation of receptors, and cofactors of proteins in the form of non-annular lipids (Huster, 2014; Posttila and Róg, 2020). The interaction of these compounds with various lipid species may lead to alterations in lipid spontaneous curvature and dynamics, membrane hydrophobic thickness and lipid packing density, and/or membrane domain structure. All these properties will in turn influence the structure and function of membrane proteins and ultimately their biological function (Soubias and Gawrisch, 2012; Brown, 2017; Posttila and Róg, 2020). For instance, the action of general anesthetics is related to either direct binding to postsynaptic ligand-gated ion channels (Nury et al., 2011) or indirectly modulating the membrane properties which affects these proteins (Cantor, 1997; Griepernau et al., 2007; Griepernau and Böckmann, 2008; Jerabek et al., 2010; Weizenmann et al., 2012). Such effects are discussed to be crucial also for other membrane proteins giving rise to various side effects of our current drugs.

The physicochemical properties of membranes and their modulation through binding of small molecules have been studied for decades using in particular spectroscopic tools (Scheidt and Huster, 2008; Mouritsen and Bagatolli, 2015; Naito et al., 2018). Spectroscopic methods have been developed to study lipid chain and headgroup conformation and dynamics as well as the lateral organization of the lipids and the membrane domain structure in complex lipid mixtures (Leftin and Brown, 2011; Cebecauer et al., 2018). In particular for the latter, solid-state ^2^H NMR spectroscopy represents a useful technique (Veatch et al., 2007; Bartels et al., 2008; Bunge et al., 2008; Engberg et al., 2020). Deuteration of individual lipids in a particular mixture allows determining the phase state of the membrane and the local environment a given lipid species experiences. Even the relative proportion of a given lipid species in a given phase can be exactly determined if it partitions into more than one phase (Stahlberg et al., 2015; Yasuda et al., 2015; Engberg et al., 2020). Furthermore, from the ^2^H NMR order parameter, the chain length and cross-sectional area of the lipid chains can be determined as well as the average number of *gauche* isomers within the chains (Petrache et al., 2000; Vogel et al., 2009; Shaikh et al., 2015). Thereby, the deuteration of lipids introduces minimal perturbation of the samples and their thermotropic phase behavior (Veatch et al., 2007; Bartels et al., 2008; Bunge et al., 2008). Furthermore, if lipids are subject to slow exchange between two phases, which occurs when the domains are large (i.e., μm size), two component ^2^H NMR spectra are observed (Veatch et al., 2007; Engberg et al., 2019). In contrast, if the domain size is small, lateral lipid diffusion is responsible for a fast exchange of molecules between the two environments allowing to detect NMR spectra consisting of only one (averaged) spectral component.

In addition to spectroscopic methods, molecular dynamics simulations have become an indispensable tool in the description of phospholipid membranes and their interaction with peptides, proteins, or drugs from the atomistic length scale to tens of nanometers and on the pico- to microsecond timescale (Pluhackova and Böckmann, 2015; Marrink et al., 2019; Chen et al., 2020). While the access to (spontaneous) lipid domain formation in simulations was initially restricted to coarse-grained methodologies [e.g., the MARTINI model (de Jong et al., 2013)] at the cost of modified thermodynamics of membranes, atomistic simulations caught up: lipid force fields (see, e.g., Pluhackova et al., 2016; Sandoval-Perez et al., 2017), improved algorithms and computer speed allow for the study of chemical (Sodt et al., 2014; Javanainen et al., 2017) and physical (Murtola et al., 2006; Kirsch and Böckmann, 2019) nanodomain formation. The combination of NMR and other experimental methods with MD simulations have yielded important insight into the structure and dynamics of lipid membranes and their interaction with small molecules (Feller et al., 1999, 2002; Sodt et al., 2014; Vogel et al., 2014, 2016; Doktorova et al., 2020).

Here, we report in detail how the small neurotransmitter serotonin (5-hydroxytryptamine, 5-HT) interacts with a raft model membrane composed of monounsaturated palmitoyl-oleoyl-phosphatidylcholine (POPC), palmitoyl-sphingomyelin (PSM), and cholesterol (Chol). 5-HT is an important neurotransmitter and signaling molecule in the human body. The serotonergic system is a major target for psychotropic drugs. 5-HT can reach very high concentrations of up to 270 mM in synaptic vesicles (Bruns et al., 2000; Balaji et al., 2005). Also efficient binding of 5-HT to lipid membranes has been reported (Peters et al., 2013; Josey et al., 2020). In particular, the influence of 5-HT on the lipid packing properties and the membrane domain structure was of interest. 5-HT has been known to partition into the lipid water interface of model membranes altering membrane structure and lipid packing (Yau et al., 1998; Peters et al., 2013). The octanol/water partition coefficient (log P) of 5-HT has been determined to be between 0.56 and 0.73 indicating its lipophilic potential (Postila et al., 2016; Turnbulla et al., 2020). Indeed, also MD simulations hinted to its membrane binding preference, 84% of the 5-HT was found membrane-bound (Postila et al., 2016). We applied a combination of experimental ^2^H NMR spectroscopy and atomistic MD simulations to describe the preference of 5-HT for differently ordered lipid membrane domains and its domain-specific influence on the membrane structure and dynamics. We used a lipid mixture consisting of POPC, PSM, and Chol at a 4:4:2 molar ratio mimicking the composition of the extracellular leaflet of eukaryotic plasma membranes.

## Materials and Methods

### Materials

Cholesterol (Chol), 1-palmitoyl-2-oleoyl-*sn*-glycero-3-phosphocholine (POPC), 1-palmitoyl-*d*_31_-2-oleoyl-*sn*-glycero-3-phosphocholine (POPC-*d*_31_), *N*-palmitoyl-D-erythro-sphingosylphosphorylcholine (PSM), *N*-palmitoyl-*d*_31_-D-erythro-sphingosylphosphorylcholine (PSM-*d*_31_), and sphingomyelin from chicken egg (egg SM) of highest quality were purchased from Avanti Polar Lipids (Alabaster, AL, United States). 5-HT and 5-HT -*d*_4_ was purchased from Merck (Darmstadt, Germany). Cholesterol-*d*_6_ was purchased from Cambridge Isotope Laboratories, Inc. (Tewksbury, MA, United States). All other chemicals were of the highest purity with the exception of organic solvents, which were of spectroscopic grade.

### Sample Preparation

Lipids were dissolved in chloroform/methanol (2:1) and 5-HT was dissolved in methanol. Aliquots of all components were mixed. Subsequently, the organic solvents were evaporated in a rotary evaporator. The lipid film was dissolved in cyclohexane followed by freezing in liquid nitrogen and overnight lyophilization, which resulted in a fluffy powder. The samples were hydrated to 50 wt% using aqueous buffer (20 mM K_2_PO_4_, 100 mM NaCl, 0.1 mM EGTA, pH 7.4) followed by 10 freeze-thaw cycles between liquid nitrogen and a 40°C water bath for equilibration. Finally, the samples were transferred to 4 mm NMR rotors and sealed for ^2^H NMR measurements.

Planar supported lipid bilayers were prepared for AFM experiments by mixing aliquots of POPC, egg SM, and Chol and dissolving them in chloroform, which was evaporated under an Argon flux and the lipid film was dried under vacuum for 24 h. The lipid film was rehydrated in water to a final concentration of 2.5 mg/ml. The lipid suspension was then vortexed vigorously and extruded using a 50 nm pore diameter polycarbonate membrane. 50 μL of this extruded lipid dispersion and 250 μL of ∼13 mM of CaCl_2_⋅2H_2_O was deposited on freshly cleaved mica previously glued on to a glass coverslip affixed to a liquid cell and incubated for 1 h at 60°C in a water bath and slowly cooled to room temperature. Samples were rinsed extensively with deionized water to remove non-fused vesicles.

### Solid-State NMR Spectroscopy

^2^H NMR spectra of the multilamellar vesicles were acquired on a Bruker 750 Avance I NMR spectrometer using a double channel solids probe equipped with a 5 mm solenoid coil operating at a resonance frequency of 115.1 MHz for ^2^H. For signal acquisition, the phase-cycled quadrupolar echo sequence (Davis et al., 1976) with two π/2 pulses of 2.5–4 μs length separated by a 30 μs delay was used. The spectral width was ±250 kHz. Measurements were carried out between 20 and 50°C with a temperature increment of 2 K. All analysis and parameters calculated from the NMR spectra was performed in programs written in the Mathcad software (MathSoft, Cambridge, MA, United States) as described before (Huster et al., 1998). ^2^H NMR lineshape simulations were performed as described in Stahlberg et al. (2015). In brief, time domain data were simulated as free induction decays (FID) for *i* superimposed Pake doublets each scaled by an order parameter *S*_*CDi*_ according to:

(1) $$F\,I\,D\,\left(t\right)=\sum_{i=1}^{N}(\left(\int_{\theta =0}^{90{}^{\circ }}d\theta \cos\left(\frac{3}{4}\chi \cdot \frac{1}{2}\left(3\cos^{2}\theta -1\right)\cdot S_{C\,D\,i}\cdot t\right)\cdot \sin\theta \right)\cdot \exp\left(t\cdot LB_{i}\cdot \pi \right)).$$

In Eq. 1, *LB* is the line broadening factor, χ = 167 kHz is the quadrupolar coupling constant for the C-^2^H bond, and θ the angle for powder averaging, which was incremented by 0.00625°. The FID was calculated as a function of time (*t*) for n data points using *t* = n ⋅ DW, with a dwell time (DW) of 1 μs.

### AFM Force Indentation

AFM force measurements (Butt and Franz, 2002; Chiantia et al., 2006; Sullan et al., 2013) were acquired using the NanoWizard II system (JPK Instruments, Berlin, Germany), mounted on an Axiovert Inverted Microscope (Zeiss, Germany). The deflection sensitivity, resonance frequencies (both in air and in water) and resultant spring constant were measured via thermal noise method. The cantilever used for all the force experiments had a resonance frequency of 10–20 kHz and a spring constant of 0.025–0.035 N/m. These values remained the same before and after the experiments. AFM experiments were performed on mica glued to the glass surface on a liquid cell, and the bilayer was hydrated till the experiment was completed. The total z-piezo displacement was 1.0 μm, and the speed both for approach and retraction were kept at 0.5 μm/s. In the non-contact region, the AFM tip approaches the top surface of the bilayer where the force remains constant. As it touches the bilayer surface at the contact point, the force gradually increases, this is followed by a sudden breakthrough of the bilayer which appears as a “kink” in the smooth force-distance curve. The value at which this occurs is the indentation force. Subsequently, the tip reaches the solid mica support. All the experiments were carried out at many different positions on the bilayer in the absence and in the presence of 5.8 mM 5-HT. All breakthrough force curves were analyzed using the JPK data processing software. The indentation force values were extracted from each approach curve, and were plotted as a histogram.

### Molecular Dynamics Simulations

Simulation setup of the different membranes and in-depth analysis of the structure and dynamics of the domains spontaneously formed are described in detail in the companion paper to this article (Bochicchio et al., 2020). In brief: we conducted 10 μs long atomistic MD simulations [CHARMM36 force field (Klauda et al., 2010)] of POPC/PSM/Chol (4/4/2) mixtures in absence and presence of 9 mol% 5-HT. Spontaneously formed disordered and ordered phases were distinguished by virtue of a Hidden Markov Model using the lipid tail order [director order parameter (Yankova et al., 2012)] as observable for the definition of the putative ordered, disordered, and intermediate ordered states.

## Results

### 5-HT Modifies the Domain Structure of the POPC/PSM/Chol (4/4/2) Mixture as Revealed by ^2^H Solid-State NMR Spectroscopy

The phase behavior of the ternary POPC/PSM/Chol (4/4/2, mol/mol/mol) mixture in the absence and in the presence of 5-HT was studied using ^2^H NMR over a temperature range from 20 to 50°C using a temperature increment of 2 K. In three independent preparations, each component of the mixture was ^2^H labeled and the respective ^2^H NMR spectra are shown in Supplementary Figure 1. Thus, the full thermotropic phase behavior of each lipid component of the mixture was recorded.

**FIGURE 1:**
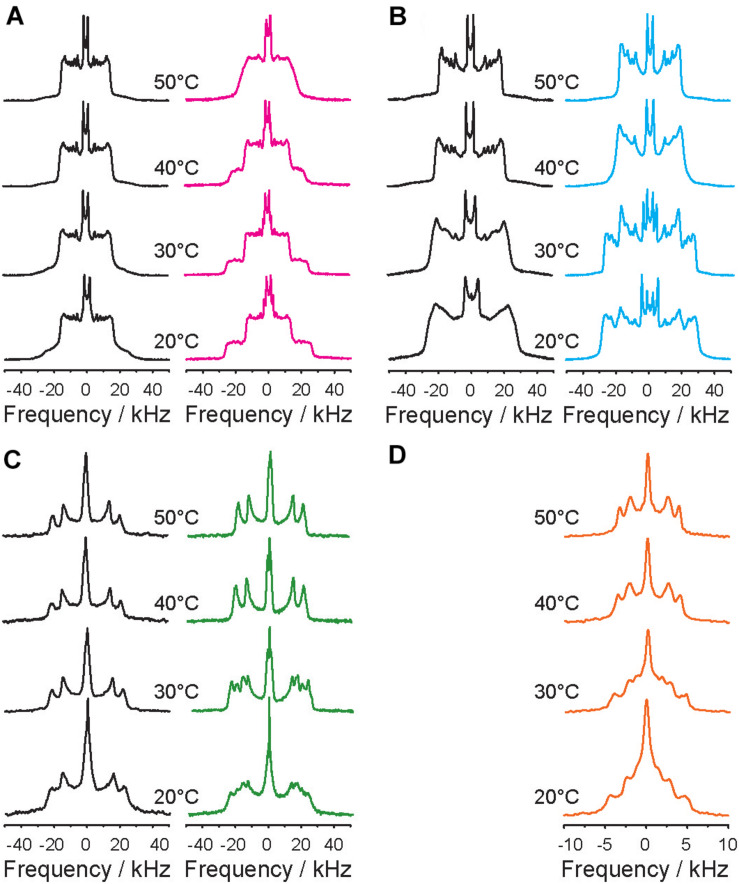
Temperature dependence of the ^2^H NMR spectra of a ternary POPC/PSM/Chol mixture (molar ratio 4/4/2) hydrated to 50 wt% aqueous buffer (K_2_PO_4_ 20 mM, 100 mM NaCl, 0.1 mM EGTA, pH 7.4) in the absence (black spectra in the left columns) and in the presence of 9 mol% 5-HT (colored spectra in the right columns) at various temperatures as indicated. **(A–D)** ^2^H NMR spectra were recorded for each component of the mixture: **(A)** POPC-*d*_31_, **(B)** PSM-*d*_31_, **(C)** Chol-*d*_6_, **(D)** 5-HT-*d*_4_.

Figure 1 displays the ^2^H NMR spectra of each ^2^H-labeled component of the POPC/PSM/Chol mixture in the absence (left spectra in black) and in the presence of 9 mol% 5-HT (right spectra in color). In the absence of 5-HT, very typical ^2^H NMR spectra characteristic of POPC forming a liquid disordered (L_d_) phase as indicated by the smaller quadrupolar splittings and PSM forming a liquid ordered (L_o_) phase as characterized by the larger quadrupolar splittings are obtained in agreement with the literature (Veatch et al., 2007; Bartels et al., 2008; Bunge et al., 2008; Soni et al., 2009; Bosse et al., 2019). The ^2^H NMR spectra of all lipid components show just one set of quadrupolar splittings for each chain methylene and methyl groups suggesting that the majority of the POPC molecules on the one hand and the PSM and cholesterol molecules on the other resides in the L_d_ and L_o_ phases, respectively (Veatch et al., 2007; Bunge et al., 2008). Previous microscopy work has demonstrated that L_*o*_ and L_d_ domains are found for this mixture (Veatch and Keller, 2003). The ^2^H NMR spectra of the Chol-*d*_6_ also feature just one quadrupolar splitting for each deuteron. These measured quadrupolar splittings agree with an upright orientation of the sterol in the membrane undergoing axially symmetric reorientations about its long axis as analyzed in detail before (Dufourc et al., 1984).

NMR investigations have also revealed that specific lipid molecules while significantly enriched in one phase are not exclusively found in just one phase (Veatch et al., 2007; Bartels et al., 2008; Bunge et al., 2008). This means that individual PSM and Chol molecules also partition into the L_d_ phase and some POPC is found in the L_o_ phase (Yasuda et al., 2015). The exchange of the lipids between the two phases is limited by lateral diffusion (Huster et al., 1998). Thus, if the domains are small, lipids can quickly exchange between the two environments. In such a case, only one averaged NMR signal is detected for each lipid species. In the terminology suggested by Feigenson and coworkers, such domains would be referred to as “nanodomains” (Usery et al., 2017). It has been estimated that on the NMR time scale, small domains would have a diameter on the order of less than about 25 nm (Huster et al., 1998). If domains are large, then the exchange of the molecules between the two environments is slow and the obtained NMR spectra consist of two contributions, one from each environment. For ^2^H NMR spectra of lipids with perdeuterated lipid chains, one observes two Pake spectra for each deuterated lipid segment (Veatch et al., 2007; Bartels et al., 2008; Bunge et al., 2008). Such large domains would be referred to as “macrodomains” (Usery et al., 2017). In the absence of 5-HT, all recorded NMR spectra only featured one component, suggesting that the domains are small such that lipids of the same species can rapidly exchange between L_o_ and L_d_ phases. Consequently, rapid lateral lipid diffusion averages the NMR signals.

The presence of 5-HT at a concentration of 9 mol% induced a drastic restructuring of the domains of the mixture. In a certain temperature range, the ^2^H NMR spectra of each component of the POPC/PSM/Chol/5-HT (4/4/2/1) mixture can be conclusively interpreted as the superposition of two NMR signals for each molecular segment (Figure 1, spectra highlighted in color). This indicates that each component of the mixture is found in two different chemical environments and that the exchange of the molecules between these environments is slow. With regard to a lipid membrane with a given domain structure, this means that the domains are very large and the lateral lipid diffusion is too slow to allow for a significant exchange between the phases. It is remarkable that the ^2^H NMR spectra of all components of the mixture showed these two contributions with unique sets of quadrupolar splittings each. Even the 5-HT molecules displayed distinct two component NMR spectra. ^2^H NMR spectra with two contributions were observed over a broad temperature range from 20–48°C for POPC, 20–38°C for PSM and cholesterol, and 20–36°C for the 5-HT. Each phase of the respective component is characterized by a unique set of quadrupolar splittings (Figure 1). At temperatures higher than 46°C, the ^2^H NMR spectra of the POPC component are exchange broadened, indicative of critical fluctuations (Veatch et al., 2007), while for PSM, Chol, and 5-HT well resolved ^2^H NMR spectra with just one set of quadrupolar splittings are detected indicating fast exchange of lipids as in the absence of 5-HT.

**FIGURE 2:**
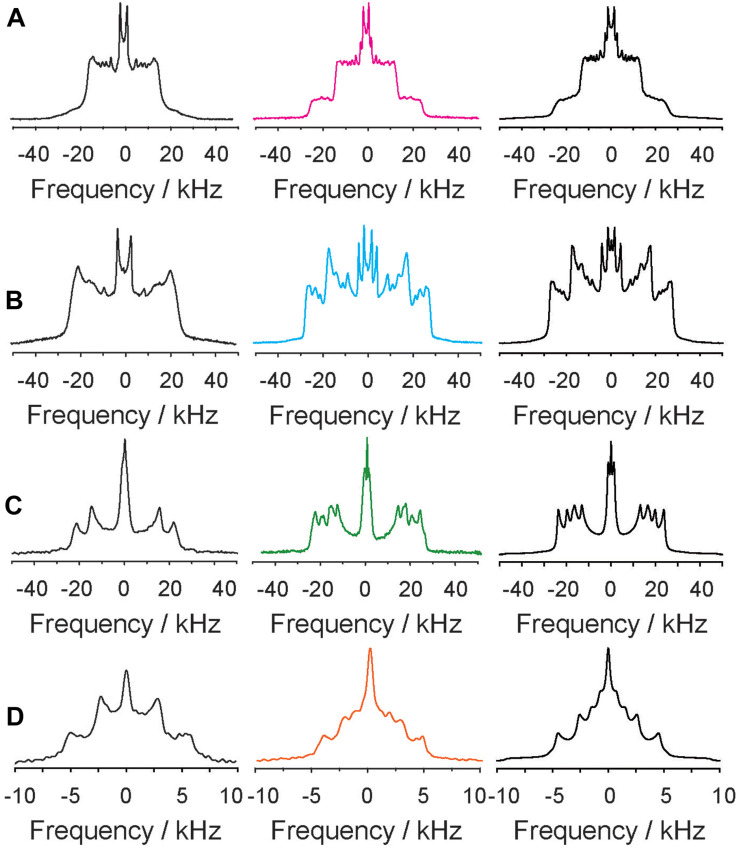
^2^H NMR spectra of deuterated POPC-*d*_31_ **(A)** PSM-*d*_31_ **(B)**, Chol-*d*_6_ **(C)**, and 5-HT-*d*_4_ **(D)** of the POPC/PSM/Chol mixture (molar ratio 4/4/2) mixture in the absence (left column) and in the presence of 9 mol% 5-HT (middle column) at a temperature of 30°C. Note that the ^2^H NMR spectrum shown in the left column of panel **(D)** was acquired for 5-HT in pure POPC membranes. The right column shows numerical simulations of the ^2^H NMR spectra.

The compositions of these phase states and the individual structural parameters of the lipid chains of each component of the mixture were analyzed in detail for the ^2^H NMR spectra recorded at 30°C. To this end, numerical simulations of the ^2^H NMR spectral lineshapes were carried out (Stahlberg et al., 2015), which are shown in Figure 2 along with the enlarged NMR spectra of each component of the mixture in the presence and in the absence of 5-HT. While the ^2^H NMR spectrum of the POPC component indicates fast exchange between molecules in small domains in the absence of 5-HT, the presence of 9 mol% (relative to all lipids) 5-HT causes a significant restructuring of the mixture. Two sets of quadrupolar splittings detected for each POPC segment suggest that the molecule partitions into two domains of relatively large size. The POPC molecules experience a significantly different chain packing in both environments. The exchange of lipids between these pools is too slow to allow for an averaging between the two environments. The two different components of the ^2^H NMR powder spectra correspond to lipids in a much more ordered and a more disordered phase (Veatch et al., 2007). For a quantitative analysis of the observed phenomena, numerical simulations of all ^2^H NMR spectra provide structural information on the individual lipid environments.

**FIGURE 3:**
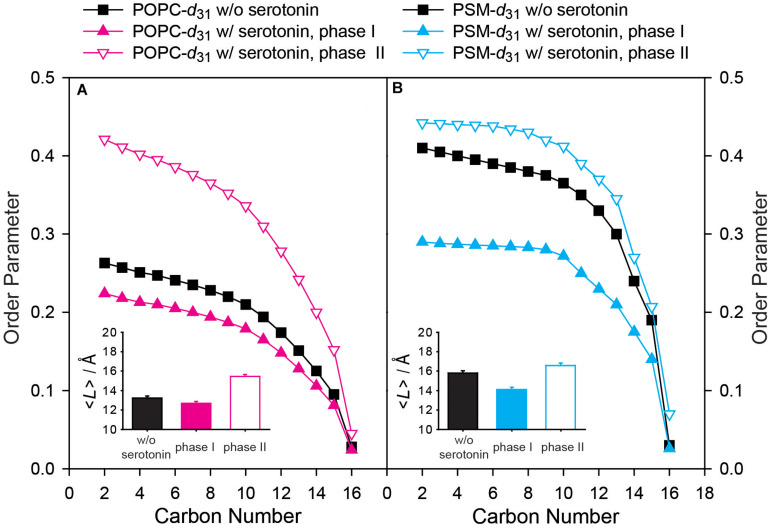
Experimental order parameter profiles and average chain length of **(A)** POPC-*d*_31_ and **(B)** PSM-*d*_31_ in a POPC/PSM/Chol (4/4/2) mixture in the absence and in the presence of 9 mol% 5-HT determined from ^2^H NMR experiments carried out at a temperature of 30°C. In the presence of 5-HT, two phases with distinctly different order parameters are observed. Phase I represents a disordered and phase II an ordered phase. The insets show the projected average chain length <*L*> of the palmitoyl chain of POPC or PSM in the absence (black bars) and in the presence of 9 mol% 5-HT (phase I filled colored bars, phase II empty colored bars). The error bars in the insets represent the error estimate of the chain length determination from ^2^H NMR spectra (Holte et al., 1996).

From these simulations, the lipid chain order parameters of the molecules in each phase could be calculated and are displayed in Figure 3. In the absence of 5-HT, the POPC molecules display an order parameter profile as typical for the L_d_ state (Bunge et al., 2008). In the presence of 5-HT, we clearly observed two lipid phases. In the first phase (referred to as phase I), the POPC molecules had somewhat lower order parameters than in the absence of 5-HT (Figure 3). In the second phase (referred to as phase II) the POPC molecules showed much higher order parameters compared to the absence of 5-HT (Figure 3). From these order parameter profiles, the projected chain lengths (⟨*L*⟩) can be estimated according to analytical models developed by the Brown laboratory (Petrache et al., 2000). These chain lengths are plotted in the insets of Figure 3. In the absence of 5-HT, the *sn*-1 chain of POPC is relatively disordered with a projected length of 13.2 Å, which means that it contains on average 5.8 *gauche* conformers as typical for an L_d_ phase. This number is calculated considering that each *gauche* defect reduces the average length of an all-*trans* chain by 1.1 Å (Feller et al., 2002). In the presence of 5-HT, the relative proportion of POPC in each individual phase can be determined from the NMR line shape simulation. 60% of the POPC is found in a more disordered state (phase I), where each acyl chain has an average length of 12.7 Å and is characterized by 6.4 *gauche* defects, while 40% of the POPC is found in a much more ordered state (phase II, ⟨*L*⟩ = 15.5 Å) with only 3.6 *gauche* defects per chain. Such parameters are characteristic of a L_o_ phase state.

Next, we analyzed the ^2^H NMR spectra of the PSM component of the mixture (Figure 2B). Characteristic ^2^H NMR spectra for the L_o_ phase state were recorded in the absence of 5-HT and the chain order parameters of PSM-*d*_31_ are shown in Figure 3B. Under these conditions, the palmitoyl chain of PSM is highly ordered (⟨*L*⟩= 15.8 Å) and contains on average 3.2 *gauche* defects. As for the case of POPC, in the presence of 5-HT, the ^2^H NMR spectra of the PSM lipids also showed the superposition of two spectral components suggesting that the lipids were organized in two environments of unique properties with only slow exchange. The chain order parameters for PSM were calculated from the ^2^H NMR spectrum and are shown in Figure 3B. Compared to the case without 5-HT, we find PSM molecules to reside within a much more disordered phase I as well as in a somewhat more ordered phase II. The disordered phase contains 37% of the PSM molecules and is characterized by lipid chains of an average length of 14.1 Å that feature on average 4.5 *gauche* defects, which is more typical of the L_d_ phase. In contrast, 63% of the PSM molecules formed the more ordered phase II which displays highly ordered chains with an average length of 16.6 Å corresponding to only 2.2 *gauche* defects characteristic for a highly ordered phase state.

The addition of 5-HT to the mixture also led to a segregation of cholesterol into two distinctly different chemical environments. We observed 22% of the cholesterol with somewhat smaller quadrupolar splittings suggesting that it partitioned into the more disordered phase I. In contrast, the majority of the Chol (78%) partitioned to the more ordered phase II. As the ratio between the individual quadrupolar splittings for each site on the molecule did not change, we conclude that the tumbling amplitude of the cholesterol in the more disordered phase I is increased (Dufourc et al., 1984), while it is slightly decreased in the more ordered phase II in agreement with the looser and tighter lipid packing in each phase, respectively.

Finally, ^2^H NMR spectra of deuterated 5-HT-*d*_4_ were analyzed. In 5-HT-*d*_4_, the two methylene groups in the sidechain of 5-HT are labeled. We obtained rather narrow ^2^H NMR spectra (Figure 2D) indicating high mobility of the molecule when partitioned into the membranes. In pure POPC bilayers, we could resolve one Pake doublet for each deuterated site (Supplementary Figure 2). In the POPC/PSM/Chol (4/4/2) membrane, again two sets of quadrupolar splittings were observed for each site characteristic for a 5-HT localization in the more disordered phase I and the more ordered phase II. From the ^2^H NMR lineshape simulations we estimated that about 28% of the 5-HT showed a more disordered ^2^H NMR spectrum while 65% of the spectral intensity expressed a slightly higher state of order. About 7% of serotonin is isotropic and presumably not bound to the membrane. Although plausible, from this analysis it cannot be concluded that 28% of the 5-HT is bound to the disordered phase I and 65% of it to the ordered phase II. Both fractions of the molecule show high mobility indicating that 5-HT moves fast undergoing large amplitude motions.

In order to investigate how these molecular changes in lipid chain packing induced by 5-HT alter the mesoscopic and mechanical properties of the membrane, we carried out AFM indentation force measurements on supported POPC/egg-SM/Chol bilayers of the same mixing ratio (Butt and Franz, 2002; Chiantia et al., 2006; Sullan et al., 2013). The force of indentation (or breakthrough force) is the force required to rupture the bilayer locally and provide the estimation of the membrane’s stiffness. We compared the breakthrough force of the bilayer in the absence and in the presence of 5.8 mM serotonin incubated for 1 h. The representative breakthrough force distribution is shown in Supplementary Figure 3. The analysis showed that 5-HT binding increases the indentation force by about 44%, which is interpreted as an increase in membrane stiffness.

Given the very intriguing impact of 5-HT on the phase behavior of the POPC/PSM/Chol (4/4/2) mixture, we also conducted the same set of experiments in equimolar POPC/PSM mixtures in the absence of cholesterol as the sterol represents the most crucial player in membrane domain formation (Brown and London, 1998). In a POPC/PSM/5-HT (5/5/1) molar mixture, we observed single component ^2^H NMR spectra for each phospholipid of the mixture at various temperatures (Supplementary Figure 4). The POPC-*d*_31_ of the mixture is found in a disordered and the PSM-*d*_31_ in an ordered state; the chain order parameters of both components are fairly different suggesting demixing of the two phospholipids (Supplementary Figure 4 and Figure 5). But the ^2^H NMR spectra of each phospholipid only show one component each. This indicates that Chol is indeed the key component for the observed alterations of the domain structure of the lipid mixture in the presence of 5-HT and 5-HT alone cannot induce the restructuring of the domains as observed for the raft mixture in presence of cholesterol.

### Analysis of the Phase State of the POPC/PSM/Chol (4/4/2) Mixture in the Absence and in the Presence of 9 mol% 5-HT Using Molecular Dynamics Simulations

**FIGURE 4:**
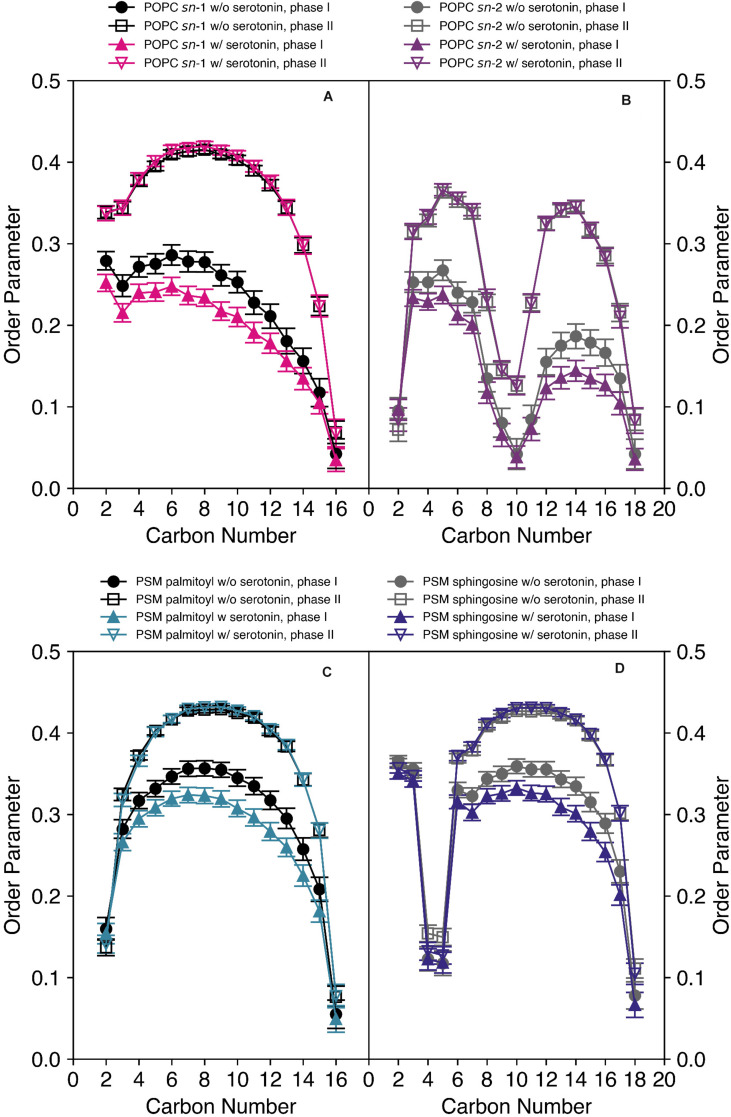
Order parameter profiles of POPC-*d*_31_ **(A,B)** and PSM-*d*_31_ **(C,D)** in a POPC/PSM/Chol (4/4/2) mixture in the absence and in the presence of 9 mol% 5-HT determined from a MD simulation at a temperature of 30°C. Upon the addition of 5-HT, an increased difference in the order parameters between the two phases was observed. I indicates the disordered and II the ordered phase or domain.

We also carried out MD simulations of the same mixture in the absence and in the presence of 5-HT, which yielded a wealth of information on the interaction of the neurotransmitter with the mixed membranes. Methods and further results of the MD simulation are discussed in detail in the companion paper to this article (Bochicchio et al., 2020). Here, we focus on the analysis of the serotonin-dependent chain order parameters of both POPC and PSM. The order parameters of either lipid species in the mixed membrane determined from the MD simulation are displayed in Figure 4.

**FIGURE 5:**
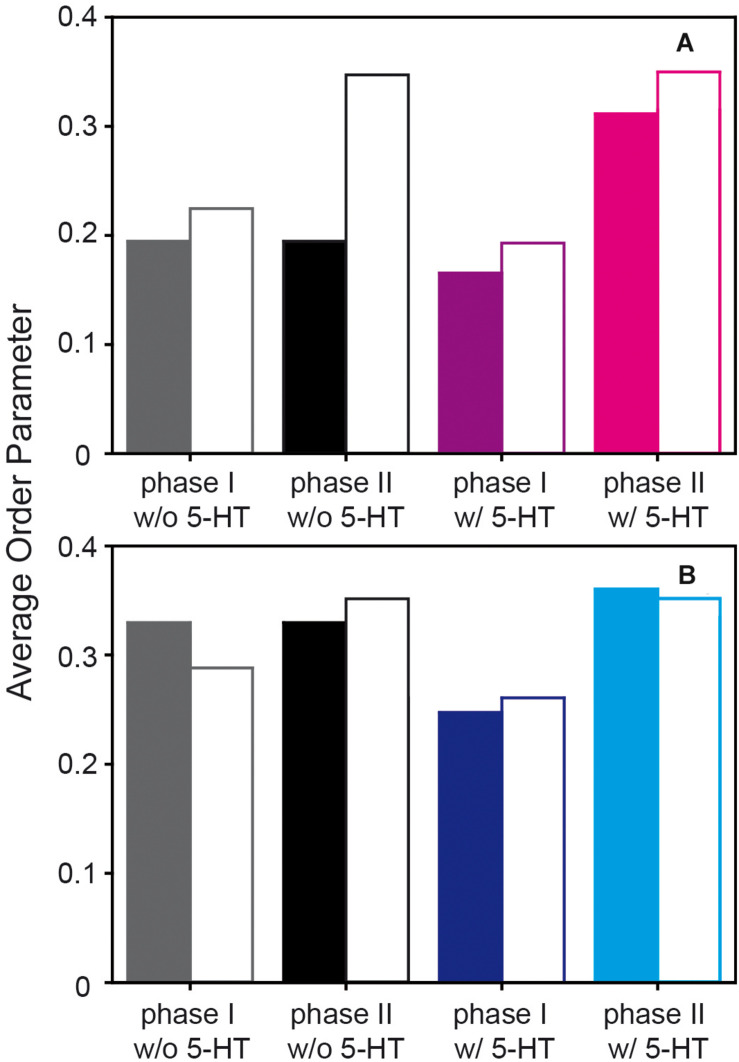
Comparison of the average order parameters of the *sn*-1 chain of POPC **(A)** and the acyl chain of PSM **(B)** in a POPC/PSM/Chol (4/4/2) mixture in the presence and in the absence of 5-HT determined by ^2^H NMR experiments (filled bars) and the MD simulations (open bars). Note that in the absence of 5-HT only one averaged lipid phase is observed experimentally and the same bars are reported.

First, it is important to discuss characteristic differences between the experimental ^2^H NMR order parameters and those determined from the MD simulations. (i) Experimentally, only the order parameters of the perdeuterated *sn*-1 chain of POPC or palmitoyl chain of PSM can be observed as only lipids with this deuteration scheme are available. In contrast, from the MD simulation, also the order parameters of the *sn*-2 chains of POPC and the sphingosine moiety of PSM are readily accessible as shown in Figures 4B,D. (ii) While the chain order parameter profiles determined by experiment gradually decrease from the glycerol backbone toward the end of the chains, the order parameter profiles determined from the MD feature a low order parameter for the C-2 segment, an order increase for the subsequent carbons toward the middle of the chain and an order decrease again toward the chain ends. The reason for this difference is the lacking assignments in the experimental ^2^H NMR spectra. Since lipids with perdeuterated chains are used in the experiment, the assignment of an order parameter to the respective carbon position in the chain is not available due to the superposition of Pake doublets in the experimental ^2^H NMR spectra. By convention (Davis, 1983), the order parameter, determined from the largest quadrupolar splitting is assigned to the C-2 position in the chain, the next lower order parameter to the C-3 position and so forth. In contrast, in the MD, the exact assignment of a given order parameter to its respective carbon atom in the chain is absolutely clear. Therefore, only the MD simulation reports the correct ordering of the individual hydrocarbon order parameters along the acyl chain. (iii) The *sn*-2 chain order parameters of the oleoyl moiety are very low in the middle of the chain due to the orientation of the double bond that results in an orientation close to the magic angle (54.7°) between the C-^2^H bond vectors and the membrane normal. This is also seen for the *trans* double bond in the sphingosine backbone of PSM providing very low order parameters for the C-4/5 segments.

Close inspection of the MD order parameter profiles revealed several interesting details. In the absence of 5-HT, POPC molecules were found in both ordered and disordered domains characterized by corresponding order parameters (Figures 4A,B). Upon the addition of 5-HT to the solvent phase of the simulation system, the order parameters of POPC in the ordered state remained relatively similar, while the POPC molecules in the disordered phase displayed significantly decreased order parameters (after the establishment of an equilibrium between membrane-bound and free 5-HT). These features were observed for both chains. For PSM, a similar situation was encountered (Figures 4C,D). In the absence of 5-HT, PSM was found in both more ordered and disordered phases. Upon addition of 5-HT, the order parameters profiles of the ordered phase II remain approximately constant while the order parameters of the disordered phase I decreased rather drastically. Again, this effect was observed both for the palmitoyl and the sphingosine chains.

For comparison of the absolute order parameters determined from experiment and from simulation illustrated in Figure 5, only the average over the *sn*-1 chain of POPC or the acyl chain of PSM could be considered due to the assignment problem of ^2^H NMR. Inspection of Figure 5 shows that the chain order parameters determined from the experiment and the simulation agreed slightly better for PSM than for POPC. Overall, the pronounced alterations that 5-HT induced on chain order were by and large reproduced in the simulation. For POPC, the experimental order parameters were generally lower than those from the MD simulation. In very good agreement with the experiment, the difference between the order parameters of phase I (disordered phase) and phase II (ordered phase) increased in presence of 5-HT. Furthermore, the MD also confirmed that even in the absence of 5-HT, each lipid species partitioned into ordered and disordered domains (phases I and II, respectively) with pronounced order differences.

## Discussion

Ternary lipid mixtures of an unsaturated phospholipid, sphingomyelin (SM) and cholesterol are subject to a complex phase behavior giving rise to a multitude of domain sizes and shapes depending on temperature, lipid chemistry, molecular mixing ratio, hydration level etc. (Baumgart et al., 2003; Rosetti et al., 2017). There is agreement that SM and Chol are predominantly found in the L_*o*_ phase, while the unsaturated phospholipid forms the L_d_ phase (Pike, 2006). However, these phases are not “pure” as SM and Chol are found as well in the L_d_ phase and the unsaturated phospholipid in the L_*o*_ state (Yasuda et al., 2015). Although these phenomena are best described by fluctuations of the lipid distribution (Veatch et al., 2007), in equilibrium a steady lipid distribution is achieved.

In the current work, we observed the striking result that the small aromatic neurotransmitter 5-HT that partitions into the lipid water interface of the membrane can drastically restructure a heterogeneous lipid mixture consisting of POPC/PSM/Chol at a 4/4/2 molar ratio. Binding of 5-HT to these membranes (i) leads to a restructuring of the domain sizes in the lipid membrane, in which (ii) the lipids form much larger domains with more pronounced ordered and disordered characteristics compared to the L_*o*_ and L_d_ states observed in the absence of 5-HT. (iii) Each component of the mixture is found to be present in both phases at different proportions. These phenomena are not induced by 5-HT-binding exclusively to one domain, as (iv) 5-HT is found to partition into both the disordered and the ordered lipid phases. These results were found at a 5-HT concentration of 9 mol% relative to the lipids in the mixture. This number corresponds to the membrane partitioning of 5-HT when lipid vesicles are incubated with 5 mM serotonin in solution, which is reasonably representative of the broad range of serotonin concentrations that physiological membranes are exposed to (S. Dey and S. Maiti, unpublished).

How can a small membrane binding neurotransmitter induce such a remarkable restructuring of the equilibrium domain structure of the ternary membrane mixture? In understanding these interesting results it is useful first to recall Gibbs phase rule which states that the number of degrees of freedom for a system with a fixed number of phases increases when the number of components of the mixture increases (Rosetti et al., 2017). As 5-HT binds to the lipid molecules, it can be considered a component of the system which consequently increases its degrees of freedom. On a more molecular level, critical phenomena at the phase boundary as well as contributions from lateral tension, line tension, bending resistance, dipole interaction, and normal pressure differences have to be considered (Baumgart et al., 2003). The line tension is pronounced to be the decisive factor that determines the size of the domains in heterogeneous membranes (Baumgart et al., 2003; Rosetti et al., 2017; Usery et al., 2017; Tsai and Feigenson, 2019). Line tension describes the interfacial energy at the edge of the membrane domains. This parameter is critically influenced by the hydrophobic mismatch between lipid chains at the interface between coexisting phases. This is exemplified by the exchange of monounsaturated PLs to di-unsaturated symmetric PCs in saturated PL/cholesterol-containing bilayers, leading to hydrophobic mismatch and therefore nanosized domains to become micro sized domains as detected in both confocal microscopy (Usery et al., 2017) and ^2^H NMR (Nyholm et al., 2019). Our experimental results show that binding of 5-HT to both the L_*o*_ and L_d_ phases alters the lipid chain order of both phospholipids rather drastically. Both our experimental and MD simulation results show that the disordered phases become more disordered. The NMR experiments further revealed a more ordered L_*o*_ phase upon binding of 5-HT (Figure 3 and Figure 5). Taken together, the hydrophobic mismatch between the lipid molecules in the two phases increases, which is supposed to increase the line tension at the phase boundary (Tsai and Feigenson, 2019; Huang et al., 2020). This effect provides the driving force for the restructuring of the domain size in the presence of 5-HT (Usery et al., 2017; Huang et al., 2020). This simple argument can explain why 5-HT binding to the lipid mixture increases the domain size as experimentally observed. Furthermore, 5-HT introduces an additional dipole moment into the membrane, which may also influence domain size (Usery et al., 2017).

On a molecular level, the question remains how neurotransmitter binding can on the one hand increase the order of the PSM in L_*o*_ phase and at the same time decrease the order of POPC in the L_d_ phase. In asking what are the molecular mechanisms by which 5-HT exerts its pronounced effects on lipid chain order, we looked into the details of 5-HT binding to the lipid molecules in either phase state. Several experimental and MD simulation studies have determined the distribution of 5-HT (Peters et al., 2013; Josey et al., 2020) or other indole derived small molecules (Yau et al., 1998) in the membrane. As observed for other small aromatic compounds (Scheidt et al., 2004; Siarheyeva et al., 2006; Weizenmann et al., 2012), 5-HT prefers an interface localization within the lipid bilayer that comprises the headgroup, glycerol, and upper chain region of the membrane in agreement with experimental studies (Dey et al., 2020; Josey et al., 2020) and MD simulations (Peters et al., 2013). Such an interface location was also confirmed in our MD simulations as discussed in detail in the companion paper (Bochicchio et al., 2020). Interestingly, in our MD simulation, a bimodal distribution of 5-HT parallel to the membrane normal was observed (Supplementary Figure 6): some of the 5-HT was found associated with the lipid headgroups and some was localized more deeply in the upper chain region of the lipid membrane (Dey et al., 2020). We analyzed if there was a correlation between the depth of 5-HT penetration into the membrane and the phase state of the respective domain in the MD data. This analysis revealed that 5-HT penetrates more deeply into disordered L_d_ membrane domains while a more superficial association of 5-HT with the lipid headgroups is primarily observed in the L_*o*_ phase (Supplementary Figure 6A). From the MD simulation of the ternary lipid mixture, we determined the number of contacts between 5-HT and the lipids in the L_*o*_ and the L_d_ phase revealing that 53.0% of the total 5-HT is associated with the L_d_ and 16.4% with the L_*o*_ phase at 30°C. However, there was a significant difference in the distribution of the 5-HT along the membrane normal: in the L_*o*_ phase, only 7.3% of the lipid contacts of 5-HT occurred with the glycerol/upper lipid chain segments while 92.7% were in contact with the lipid headgroups. In contrast, in the L_d_ phase, 29.2% of the 5-HT contacts were observed with the glycerol/upper chain region and 73.1% with the headgroup. This increased number of contacts of 5-HT with the glycerol/upper chain segments of L_d_ phase lipids and with the headgroup of L_*o*_ phase lipids was confirmed by MD simulations of 5-HT binding to pure L_*o*_ and L_d_ phases, respectively [Supplementary Figures 6B,C; for details see companion paper (Bochicchio et al., 2020)].

Our results suggest that 5-HT, bound to the glycerol upper chain region of the L_d_ phase lipids, causes a decrease in the lipid chain order parameters. 5-HT molecules penetrate the glycerol/upper chain segments and thus decrease the packing density in the membrane providing more free volume in the lower chain region which is occupied by larger amplitude motions of the chain segments causing lower order parameters in the more disordered phase. In contrast, 5-HT that is bound to the lipid headgroup region may have a tendency to decrease the repulsive forces between the lipid headgroups. 5-HT may intercalate between negatively charged phosphate groups thus leading to an increase in lipid chain packing density expressed by higher chain order parameters of the more ordered phases. Such resulting area compression in lipid membranes has been reported previously for membranes interacting with anionic polyelectrolytes in the presence of divalent cations or positively charged lipids (Huster et al., 1999, 2000). Also, interactions between 5-HT and the lipid’s phosphate groups have been described in previous MD simulations (Peters et al., 2013). Taken together, the formation of the more disordered phase I and the more ordered phase II of the ternary mixture observed in the presence of 5-HT is likely the consequence of the different insertion depths into and binding strengths to the L_*o*_ and L_d_ membrane phases as analyzed from corresponding MD simulations of 5-HT binding to pure L_d_ (POPC/PSM/Chol, molar ratio 69/23/8) and L_*o*_ (POPC/PSM/Chol, molar ratio 8/61/31) ternary membrane compositions (see Supplementary Figure 6). For the L_*o*_ phase, two localized regions were found with maxima either inside the membrane headgroup region or in a diffusive adsorption layer on the membrane surface (Supplementary Figure 6). In contrast, only one broader binding region embedded more deeply within the membrane is seen for the L_d_ phase. In turn, the increased acyl chain order within phase II is suggested to be related to the preferred 5-HT binding to the lipid headgroup region as well as to the increased domain sizes that allowed to distinguish different phases for PC lipids within the NMR spectra only in the presence of 5-HT. However, while the simulations do show a decreased ordering within the disordered phase and a more distinguished phase separation (see also companion manuscript), a direct effect of 5-HT on the acyl chain order within the ordered phase could not be seen. This observation is possibly related to the limited size of the studied membrane patches as well as slow processes in phase formation beyond the simulation time scale of 10 μs. It appears that the 5-HT-induced increase of the lipid chain order, and the intimately coupled phase separation well beyond a length scale of ten nanometer, are relevant for rendering the membrane stiffer in the presence of 5-HT as observed in the AFM measurements (Supplementary Figure 3).

Phase separation phenomena as observed in lipid membranes and raft models are always related to both the temperature of a specific preparation relative to the main phase transition temperature of the lipid species in the mixture and its specific cholesterol content (Veatch et al., 2007; Sodt et al., 2014). In that regard, the ^2^H NMR spectra of DOPC/DPPC-*d*_62_/Chol mixtures at varying compositions, reported by the Gawrisch and Keller labs, are particularly informative (Veatch et al., 2007). For instance, phase pure ^2^H NMR spectra of the L_*o*_ and the L_d_ phases at 20°C were reported for 4/1 DOPC/DPPC-*d*_62_ mixtures in the presence of 40 or 15% Chol, respectively. Furthermore, ^2^H NMR spectra indicative L_d_/L_*o*_ phase coexistence as observed here in the presence of 5-HT were reported for an equimolar DOPC/DPPC-*d*_62_ mixture in the presence of 15% Chol. In addition, L_d_/L_*o*_ phase coexistence by ^2^H NMR has also been observed in SM/Chol bilayers with different di-unsaturated phospholipids, e.g., 14:1, 18:1, 20:1-PC at the same molar lipid ratio of unsaturated PC/SM/Chol 4/4/2 as used in our study (Nyholm et al., 2019). We did not observe any 5-HT-induced alteration of the phase composition of POPC/PSM membranes in the absence of Chol (Supplementary Figure 4 and Figure 5), so it is clear that cholesterol is the major factor in the observed phenomena. This is related to the preferential interaction of cholesterol with saturated chains and the fact that the cone shaped Chol strongly influences membrane curvature. Interestingly, simulations performed at temperatures between 21 and 30°C of the POPC/PSM/Chol mixture further suggest a serotonin-induced change in domain composition: Addition of 5-HT led to a cholesterol content of the disordered phase decreased by 14–29% (see Table 3 in Bochicchio et al., 2020). Thus, the differential 5-HT binding results in a modified interaction network at the membrane interface leading to the observed remodeling of the membrane domain composition, size, and structure. By 5-HT binding to the upper chain region, the favorable interactions between Chol and the saturated lipid chains (Huster et al., 1998; Almeida et al., 2005; Tsamaloukas et al., 2006; Frazier et al., 2007; Scheidt et al., 2013) are perturbed potentially contributing to the observed restructuring of the membrane domains.

In conclusion, we report that the small neurotransmitter 5-HT binds to biologically relevant membrane models and induces a significant restructuring of its domain size. By modulating membrane properties, sorting of membrane proteins may be influenced impacting their structure and dynamics (Huang et al., 2020). For G protein-coupled receptors, secondary structure adaptation to the membrane hydrophobic thickness (Soubias et al., 2008) as well as alterations in the dynamical behavior in various membrane compositions (Thomas et al., 2015) have been reported. Essential membrane properties such as chain length and unsaturation critically influence the function of rhodopsin (Litman and Mitchell, 1996). Thus, 5-HT and small molecule binding to lipid membranes can have a profound influence on the function of non-cognate receptors by regulating their conformational equilibrium. The study suggests that a complex interplay between the lipids of the membrane with neurotransmitters binding from the aqueous phase can alter the function of membrane proteins through alterations in membrane hydrophobic thickness and membrane domain size in the biological system (Postila et al., 2016).

## Data Availability Statement

The original contributions presented in the study are included in the article/Supplementary Material, further inquiries can be directed to the corresponding authors.

## Author Contributions

SM and DH designed the research. OE did all experimental work using NMR spectroscopy. AG and SD did the AFM experiments. AB and RB carried out MD simulations. OE and DH analyzed the ^2^H NMR spectra and performed lineshape simulations. AG, SD, and SM analyzed the AFM measurements. AB, AFB, and RB analyzed the MD simulations. DH wrote the manuscript with contributions from all coauthors. All authors contributed to the article and approved the submitted version.

## Conflict of Interest

The authors declare that the research was conducted in the absence of any commercial or financial relationships that could be construed as a potential conflict of interest.
