# Supplementary material for: Serotonin Alters the Phase Equilibrium of a Ternary Mixture of Phospholipids and Cholesterol
Source: Front Physiol. 2020 Oct 23;11:578868. doi: 10.3389/fphys.2020.578868 (PMC7645218; doi:10.3389/fphys.2020.578868)
Supplement: Supplementary file 1 [file Data_Sheet_1.docx]

Supporting Information

**Serotonin Alters the Phase Equilibrium of a Ternary Mixture of Phospholipids and Cholesterol**

Oskar Engberg,^1^ Anna Bochicchio,^2^ Astrid F. Brandner^2^,Ankur Gupta,^3^ Simly Dey,^3^ Rainer Böckmann,^2^ Sudipta Maiti,^3,^* and Daniel Huster^1,3,^*

^1^Institute for Medical Physics and Biophysics, University of Leipzig, Härtelstr. 16-18, D-04107 Leipzig, Germany

^2^Computational Biology, Department of Biology, Friedrich Alexander University, Staudtstr. 5, D-91058 Erlangen, Germany

^3^Department of Chemical Sciences, Tata Institute of Fundamental Research, Homi Bhabha Road, Colaba, Mumbai, 400 005, India


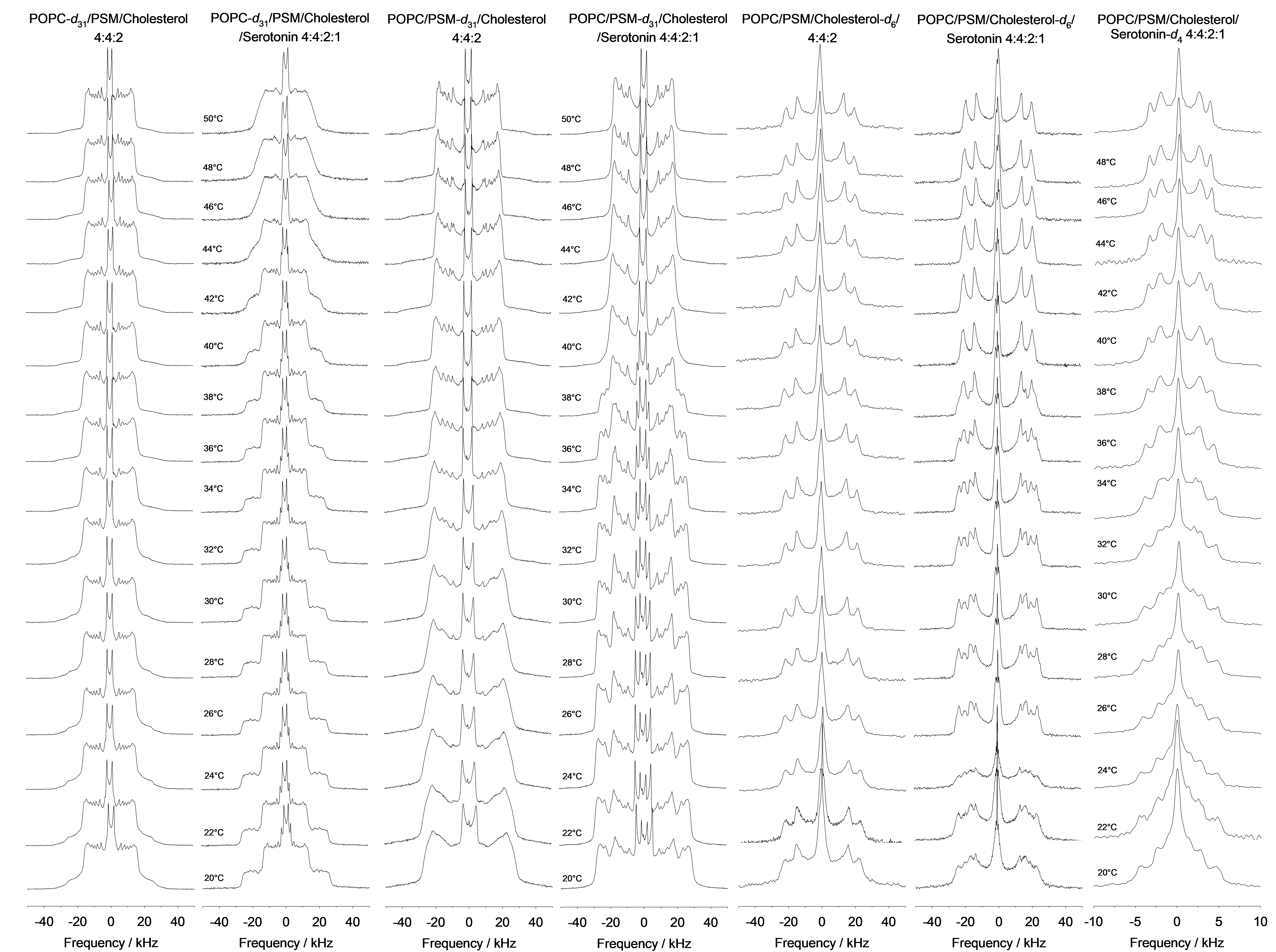


**Figure S1.** Temperature dependence of the ^2^H NMR spectra of a ternary POPC/PSM/Chol mixture (molar ratio 4/4/1) hydrated to 50 wt% aqueous buffer (K_2_PO_4_ 20 mM, 100 mM NaCl, 0.1 mM EGTA, pH 7.4) in the absence and in the presence of 9 mol% 5-HT at various temperatures as indicated.


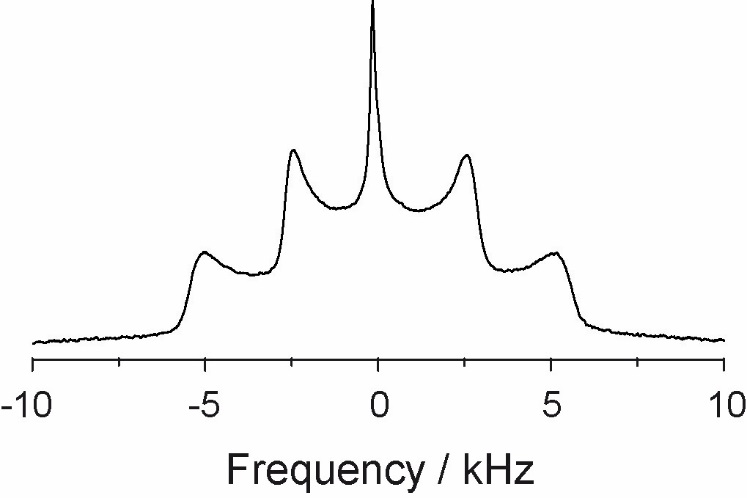


**Figure S2.** ^2^H NMR spectrum of 5-HT-*d*_4_ in POPC membranes at a temperature of 30°C.

**A**

**B**

**Figure S3.** Effect of serotonin on the mechanical properties of a ternary POPC/eggSM/Chol supported mixture (molar ratio 4/4/1). A) Representative breakthrough force distribution analysis in the absence (black) and in the presence of 5.8 mM5-HT. B) Increase of the breakthrough force of the supported membrane in the presence of 5.8 mM 5-HT (n = 2400 force traces). Error bar represent standard error of the mean.


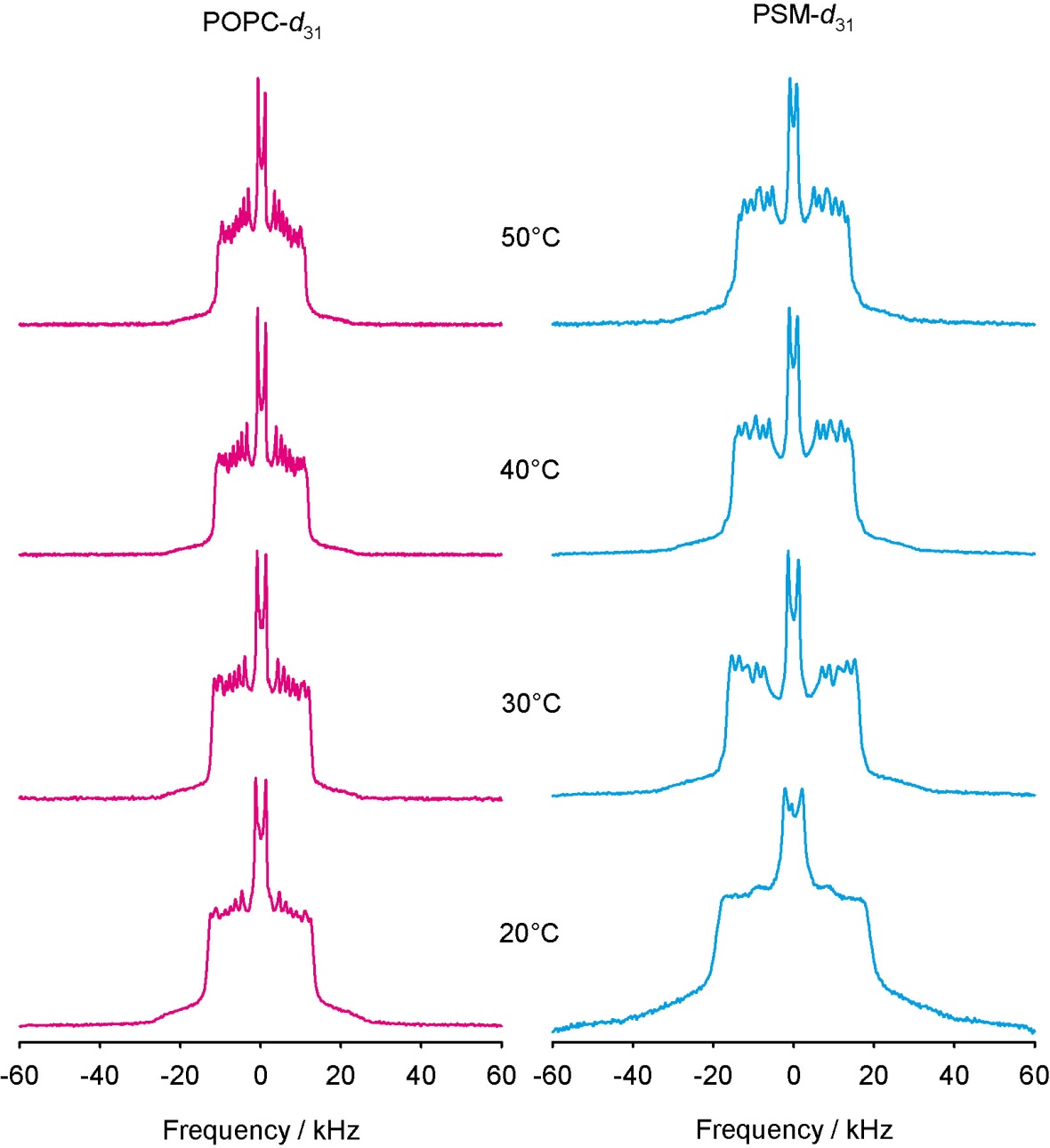


**Figure S4.** Temperature dependence of the ^2^H NMR spectra of a POPC/PSM mixture in the presence of 5-HT (molar ratio 5/5/1) hydrated to 50 wt% aqueous buffer (K_2_PO_4_ 20 mM, 100 mM NaCl, 0.1 mM EGTA, pH 7.4).


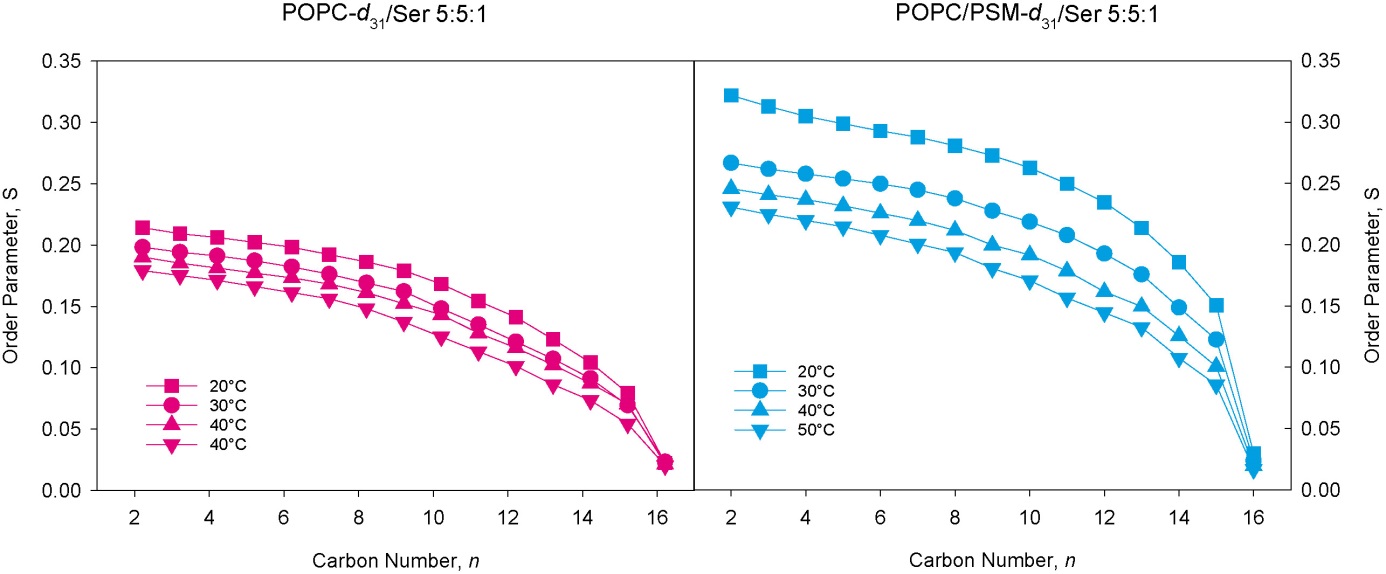


**Figure S5** Temperature dependence of the order parameter profiles of POPC-*d*_31_ (left) and PSM-*d*_31_ (right) in a POPC/PSM/5-HT (5/5/1) mixture i determined from ^2^H NMR experiments carried out at various temperatures.

**
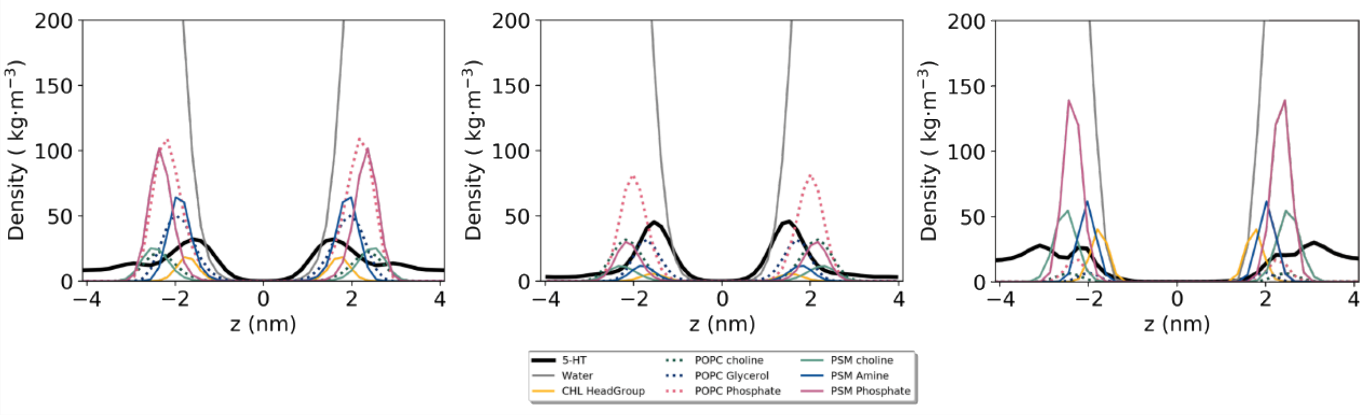
**

**Figure S6.** Distribution profiles of 5-HT and various lipid segments determined from the MD simulations of a ternary POPC/PSM/Chol/5-HT mixture (molar ratio 4/4/1/1) (A) and in membrane mixtures forming an ld phase (POPC/PSM/Chol, 69/23/8, B) or lo phase (POPC/PSM/Chol, 8/61/31, C) at a temperature of 30°C.
